# miR-205-5p inhibits human endometriosis progression by targeting ANGPT2 in endometrial stromal cells

**DOI:** 10.1186/s13287-019-1388-5

**Published:** 2019-09-23

**Authors:** Chen-Fei Zhou, Min-Juan Liu, Wei Wang, Sha Wu, Yu-Xin Huang, Guo-Bin Chen, Li-Min Liu, Dong-Xian Peng, Xue-Feng Wang, Xu-Zi Cai, Xiao-Xuan Li, Wan-Qin Feng, Ying Ma

**Affiliations:** 1 Department of Obstetrics and Gynecology, The First Affiliated Hospital of Guangzhou Medical University, Guangzhou, 510120 China; 2 Department of Obstetrics and Gynecology, Zhujiang Hospital of Southern Medical University, No.253, Middle Gongyeda Road, Haizhu District, Guangzhou, 510280 China; 3 Department of Immunology/Guangdong Provincial Key Laboratory of Proteomics, School of Basic Medical Sciences, Southern Medical University, Guangzhou, 510515 China; 4 Department of Obstetrics and Gynecology, Shenzhen Maternal and Child Healthcare Hospital of Southern Medical University, Shenzhen, 518028 China

**Keywords:** Endometriosis, miR-205-5p, Endometrial stromal cells, ANGPT2

## Abstract

**Background:**

miRNA expression profiles in ectopic endometrium (EC) serving as pathophysiologic genetic fingerprints contribute to determining endometriosis progression; however, the underlying molecular mechanisms remain unknown.

**Methods:**

miRNA microarray analysis was used to determine the expression profiling of EC fresh tissues. qRT-PCR was performed to screen miR-205-5p expression in EC tissues. The roles of miR-205-5p and its candidate target gene, angiopoietin-2 (ANGPT2), in endometriosis progression were confirmed on the basis of both in vitro and in vivo systems. miR-205-5p and ANGPT2 expression were measured by in situ hybridization and immunochemistry, and their clinical significance was statistically analysed.

**Results:**

miR-205-5p was screened as a novel suppressor of endometriosis through primary ectopic endometrial stromal cell migration, invasion, and apoptosis assay in vitro, along with endometrial-like xenograft growth and apoptosis in vivo. In addition, ANGPT2 was identified as a direct target of miR-205-5p through bioinformatic target prediction and luciferase reporter assay. Re-expression and knockdown of ANGPT2 could respectively rescue and simulate the effects induced by miR-205-5p. Importantly, the miR-205-5p-ANGPT2 axis was found to activate the ERK/AKT pathway in endometriosis. Finally, miR-205-5p and ANGPT2 expression were closely correlated with the endometriosis severity.

**Conclusion:**

The newly identified miR-205-5p-ANGPT2-AKT/ERK axis illustrates the molecular mechanism of endometriosis progression and may represent a novel diagnostic biomarker and therapeutic target for disease treatment.

**Electronic supplementary material:**

The online version of this article (10.1186/s13287-019-1388-5) contains supplementary material, which is available to authorized users.

## Background

Endometriosis is a common and multifactorial gynaecologic condition characterised by the presence of endometrial-like tissue in aberrant locations outside the uterus. Endometriosis affects an estimated 10 to 15% of women of reproductive age worldwide [1]. The disease has multiple manifestations, such as dysmenorrhoea, chronic pelvic pain, infertility, and cancerous lesions, and it can severely affect the quality of patients’ life [2, 3]. The current gold standard for the diagnosis of endometriosis is laparoscopic surgery [4]. It is unlikely that reproductive-age women would undergo such an invasive surgery when they can choose temporarily diminish pain symptoms by other treatments. However, none of the currently available techniques, including imaging methods or laboratory-developed platforms, is a suitable replacement for laparoscopy [5, 6]. Therefore, it is urgent to better understand the molecular mechanism underlying the endometriosis progression and to identify novel and more efficient diagnostic predictors for accurate identification of the disease, so that the optimal therapeutic strategies can be found.

miRNAs are small non-coding RNAs, approximately 20 to 24 nucleotides in length, that regulate target gene expression at the posttranscriptional level by pairing to 3′ untranslated regions (UTRs) of mRNAs and triggering RNA degradation or translational suppression [7]. These miRNAs are involved in multiple biological processes, such as cellular migration, invasion, and apoptosis [8, 9]. As a transcriptional regulator, aberrant expression of miRNAs usually leads to dysregulated expression of target genes that are involved in initiation and progression of various diseases including endometriosis, suggesting that they are excellent candidate biomarkers and potential therapeutic targets [10–12]. However, miRNA profiling of ectopic endometrium reflecting endometriosis progression is still unclear.

To address this problem, we performed the current study to investigate the underlying molecular mechanisms for differentially expressed miRNAs of ectopic endometrium in endometriosis progression, as well as its clinical relevance, to explore the potential clinical applications in diagnosis and therapy.

## Materials and methods

### Clinical specimens

Ectopic endometrial tissues and serum were collected from patients with endometriosis, and normal endometrial tissues and serum from hysterectomy in patients with grade II–III cervical intraepithelial neoplasia or uterine leiomyoma. The pathological diagnosis was performed preoperatively and confirmed postoperatively. All of the clinical specimens were obtained from the Department of Gynecology of Zhujiang Hospital (Guangzhou, People’s Republic of China) between 2006 and 2012 according to the ethical and legal standards. Patients receiving hormone treatment or those with concurrent malignancies were excluded. The characteristics of the patients with endometriosis are described in Additional file 1: Table S1.

### miRNA microarray

Agilent miRNA microarray 21.0 for normal endometrium (*n* = 3) and ectopic endometrium (*n* = 3) was established by Guangdong Longsee Biomedical Corporation and analysed by GeneSpringGX software 11.0 (Agilent).

### Isolation of primary endometrial stromal cells and cell culture

Primary normal and ectopic endometrial stromal cells were isolated as described previously [13]. Briefly, ectopic endometrial tissues were minced and digested in phosphate-buffered saline (PBS) containing collagenase (1 mg/mL, 15 U/mg) and 1% penicillin/streptomycin for 60 min in an orbital shaker at 37 °C. The obtained homogenous cell suspension were plated in T25 cell culture flasks (Corning) and cultured in Dulbecco’s modified Eagle medium (DMEM)/Ham’s F12 (1:1) with 10% foetal bovine serum (FBS, Gibco) and bio-antibiotics (100 IU/mL penicillin and 100 μg/mL streptomycin). Cellular purities were examined by immunofluorescence staining for vimentin (ab8978, Abcam) and cytokeratin (ab76126, Abcam) antibodies. Primary endometrial stromal cells were used between passages 3 and 5.

### Stable transfection with lentivirus

Lentivirus containing an miR-205-5p overexpression sequence and its negative control were all purchased from GeneChem Inc. Primary ectopic endometrial stromal cells were transfected with lenti-miR-205, and polyclonal cells expressing green fluorescent protein signals were selected for further experiments by flow cytometer.

### Transient transfection with oligonucleotides and plasmids

The miR-205-5p mimic and its negative control, or miR-205-5p inhibitor and its negative control, were designed and cloned by RiboBio Inc. The ANGPT2 coding sequence (without 3′-UTR) was cloned into pCDNA3.1(+)-Vector (Invitrogen). The empty vector was used as a blank control. siVASH1 and its negative control siRNA were designed and synthesised by GenePharma Inc. Lipofectamine 2000 Reagent (Invitrogen) was then used to transfect miR-205-5p mimic and inhibitor, plasmid-ANGPT2, and siANGPT2 according to the manufacturer’s protocol. For RNA extraction and western blot and in vitro functional assays, cells were used 48 h after transfection. The sequence of siANGPT2 and siRNA are shown in Additional file 1: Table S2.

### RNA extraction and quantitative reverse transcriptase polymerase chain reaction

RNA was extracted from clinical specimens, serum samples, primary cells, and xenografted lesions by the miRNeasy Mini kit (Qiagen, 217004). qRT-PCR was performed according to the protocol of our previous study [14]. Specific primer sets for miRNAs and U6 were purchased from RiboBio Inc. The expression of miRNAs and mRNAs was normalised to U6 and glyceraldehyde-3-phosphate dehydrogenase (GAPDH), respectively. The primer sequences are shown in Additional file 1: Table S2.

### Immunohistochemistry

Clinical specimens and xenografted lesions were subjected to immunohistochemistry (IHC) analysis as described previously [15]. The primary antibodies were anti-ANGPT2 (ab56301, Abcam) and anti-Annexin V antibody (ab14196, Abcam). The secondary antibodies were horseradish peroxidase-conjugated anti-rabbit immunoglobulin-G antibody (ab6721, Abcam).

### In situ hybridisation

In situ hybridisation (ISH) was performed as described previously [16]. Briefly, after incubation with H_2_O_2_, the tissue sections were treated with pepsin at 37 °C for 2 min, washed, and prehybridised for 4 h at 37 °C. Hybridisation with digoxygenin-labelled miR-205-5p LNA probes (Exiqon) occurred at 37 °C overnight. The sections were then washed at 37 °C and incubated with biotinylated mouse anti-digoxin for 1 h at 37 °C. Staining was visualised by adding 3,3-diaminobenzidine and counterstaining with haematoxylin.

### Staining assessment

The IHC- and ISH-stained tissue sections were reviewed and scored separately by two independent pathologists. For semi-quantitative evaluation of ANGPT2, Annexin V, and miR-205-5p expression in tissue sections, the German Immuno-Reactive Score was applied as previously described [17]. The immunohistochemical score was calculated by combining the proportion of positively stained cells and the intensity of staining. The staining intensity was rated on a scale of 1 to 4, with 1 representing no staining, 2 weak staining, 3 moderate staining, and 4 strong staining. No staining was scored as 0; staining of 1 to 10% as 1; 11 to 50% as 2; 51 to 80% as 3; and 81 to 100% as 4. The raw data were converted to IHS by multiplying the quantity score and the intensity score. Tissues with an IHS of 4 or greater were defined as high expression; tissues with a IHS of less than 4 were defined as low expression.

### Animal models

Female nude mice (6 weeks old) were purchased from the Experimental Animal Center, Southern Medical University (Guangzhou, People’s Republic of China). The studies were approved by the Institutional Animal Research Ethics Committee of Southern Medical University. The endometriosis mouse model was established in the Experimental Animal Center of Southern Medical University as described previously [10]. Briefly, 1 × 10^7^ ectopic endometrial stromal cells mixed with gland cells (1:1) were subcutaneously injected into the flanks of each nude mice (*n* = 6, per group). Lesion size (mm^3^) was measured every 4 days and calculated by the following formula: volume = (width)^2^ × length/2. The mice were euthanised 30 days after the injection of human ectopic endometrial cells to evaluate the extent of endometriosis.

### Western blot assay

Western blot assay was performed as in our previous study [14]. The detailed antibody information is provided in Additional file 1: Table S3.

### Other methods

Luciferase reporter assay, wound healing assay, ELISA assay, Transwell invasion assay, and apoptosis assay were performed according to the manufacturers’ protocols. Detail information is described in Additional file 2: Supplemental materials and methods.

### Statistical analysis

SPSS V.13.0 software was used for statistical analysis. Data are expressed as the mean ± standard deviation (SD). A two-tailed Student *t* test or one-way analysis of variance (ANOVA) was used for comparisons among the groups. The Fisher or chi-square test was applied for categorical variables. Partial correlations were applied in the multivariate correlation analysis. The Cox regression model was used for the univariate and multivariate analysis. Differences were considered to be statistically significant when *P* < 0.05.

## Results

### Identification of miR-205-5p as a negatively pathologic miRNA in endometriosis

**Fig. 1:**
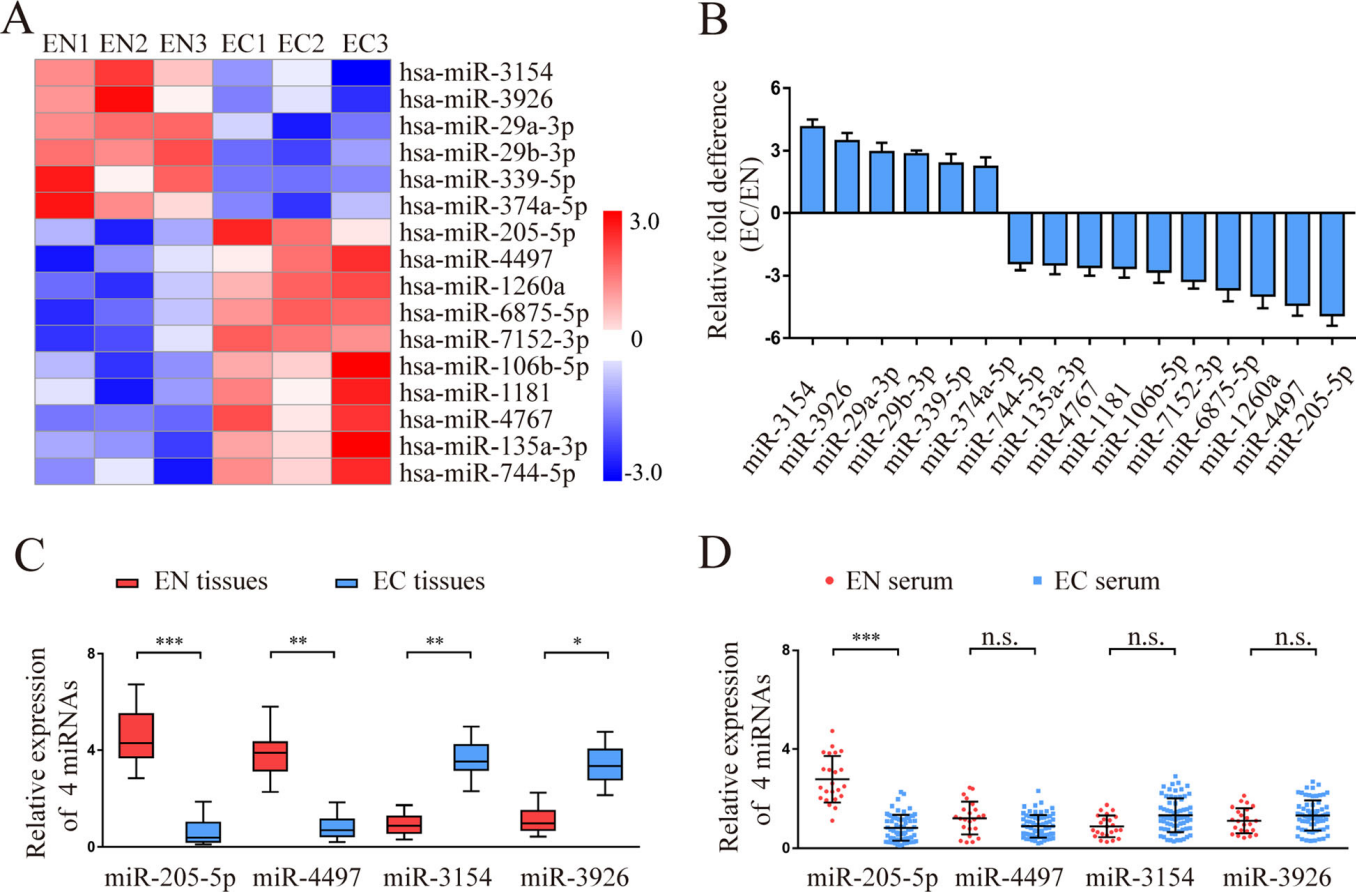
Identification of miR-205-5p as a negatively pathologic miRNA in endometriosis. **a** The different miRNA expression profiles between the EN (*n* = 3) and EC (*n* = 3) groups were analysed by miRNA microarray. The heatmap diagram shows that the representative miRNAs were significantly associated with endometriosis. EN1, EN2, and EN3 indicate 3 normal endometria; EC1, EC2, and EC3 indicate 3 ectopic endometria. **b** The levels of 16 differentially expressed miRNAs in the endometrium used for microarray analysis were analysed by qRT-PCR. **c**, **d** The levels of miR-205-5p, miR-4497, miR-3154, and miR-3926 were validated by qRT-PCR in additional tissues and serum from the EN (*n* = 23) and EC (*n* = 68) groups. EN, normal endometrium; EC, ectopic endometrium. Error bars represent the mean ± SD of 3 independent experiments. n.s., not significant; *P < 0.05; **P < 0.01; ***P < 0.001

To identify the differences in miRNAs for endometriosis, miRNA expression profiles of normal endometrium (EN) (*n* = 3) and ectopic endometrium (EC) (*n* = 3) were analysed by Agilent miRNA microarray 21.0. Using a 2-fold change and *P* < 0.05 as the threshold cut-off, we found that 16 miRNAs were significantly different between EN and EC (Fig. 1a). The differential expression of 16 miRNAs was verified by qRT-PCR in the same tissues used for microarray analysis. The results showed that a 4.89 ± 0.51-fold lower level of miR-205-5p, 4.37 ± 0.53-fold lower level of miR-4497, 4.13 ± 0.38-fold higher level of miR-3154, and 3.46 ± 0.39-fold higher level of miR-3926 were examined in EC compared with EN (Fig. 1b). miR-205-5p showed the greatest downregulation among the 4 representative miRNAs, suggesting that miR-205-5p may be the best candidate for further study.

To further confirm the statistical significance of the 4 representative miRNAs (miR-205-5p, miR-4497, miR-3154, miR-3926), additional tissues and serum sample from the EN (*n* = 23) and EC (*n* = 68) groups were analysed by qRT-PCR. Compared with the EN group, a significantly lower level of miR-205 was detected in both tissues and serum samples of the EC group (Fig. 1c, d). Taken together, these results suggested that low levels of miR-205-5p expression may be associated with endometriosis progression.

### miR-205-5p suppressed migration and invasion but promoted apoptosis of endometriosis-derived endometrial stromal cells in vitro

To investigate the functional difference between EN and EC stromal cells, we isolated two primary ectopic endometrial stromal cells, EC109 and EC520, and two primary normal endometrial stromal cells, EN211 and EN307. The immunofluorescence staining showed that both vimentin and cytokeratin were expressed in EC and EN stromal cells (Additional file 3: Figure S1A). Additionally, in the presence of E2 plus medroxyprogesterone acetate (MPA), the cells transformed from a spindle-shaped appearance to large polygonal-shaped cells and significantly increased PRL secretion (Additional file 3: Figure S1B & C), indicating that the induced endometrial stromal cells were successfully differentiated in vitro. Collectively, these results were consistent with previous studies [18, 19], suggesting that the isolated EC109, EC520, EN211, and EN307 cells were endometrial stromal cells.

To further explore the suppressive role of miR-205-5p in endometriosis in vitro, we constructed a lentiviral vector expressing miR-205-5p and established miR-205-5p-overexpressing EC109 and EC520 cells after lentivector transfection (Additional file 4: Figure S2A). More than 500-fold increase in miR-205-5p expression was examined in Lenti-miR-205-5p-transfected EC109 and EC520 cells compared with the negative control (NC) group (Additional file 4: Figure S2B). Despite very low endogenous expression of miR-205-5p in EC109 and EC520 cells (Additional file 4: Figure S2C), miR-205-5p inhibitors were transiently transfected into two cells. However, no significant difference in the expression of miR-205-5p was observed between the miR-205-5p inhibitors group and the NC group by qRT-PCR (Additional file 4: Figure S2D).

**Fig. 2:**
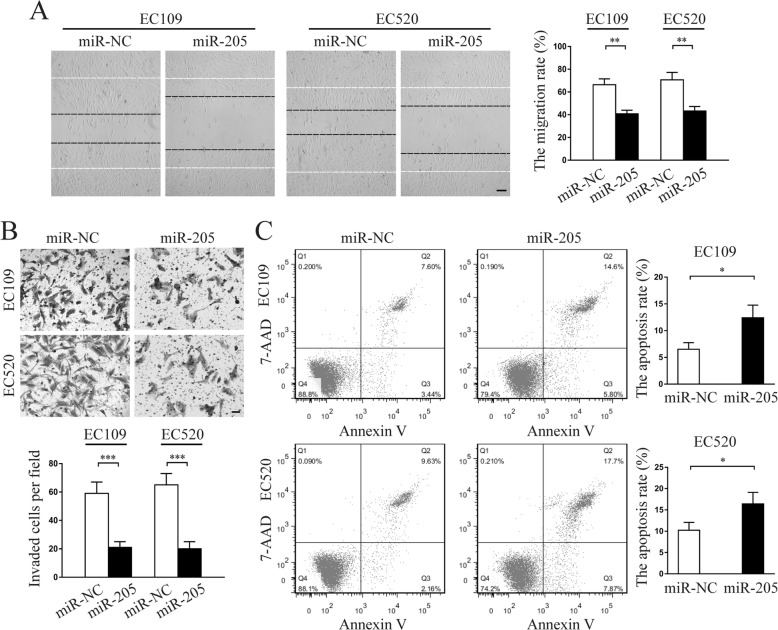
miR-205-5p suppressed migration and invasion but promoted apoptosis of EC109 and EC520 in vitro*.* **a** Representative micrographs of wound healing assay in miR-205-5p-overexpressing EC109 and EC520 compared with NC were shown. Images were acquired at 0 (white dotted line) and 48 h (black dotted line). Average migration rate per field was calculated. Scale bar, 20 μm. **b** Representative micrographs of Transwell invasion assay in miR-205-5p-overexpressing EC109 and EC520 compared with NC were shown. Average invasive cells per field were calculated. Scale bar, 50 μm. **c** Representative micrographs of apoptosis assay in miR-205-5p-overexpressing EC109 and EC520 compared with NC were shown. Average apoptosis rate per time was analysed. miR-205, miR-205-5p. Error bars represent the mean ± SD of three independent experiments. *P < 0.05; **P < 0.01; ***P < 0.001

Wound healing assay demonstrated that miR-205-5p upregulation markedly weakened the migratory abilities of both EC109 and EC520 cells compared with those of control cells (Fig. 2a). Transwell invasion assay showed that enforced expression of miR-205-5p significantly reduced the invasive activities of both EC109 and EC520 cells (Fig. 2b). In contrast, the apoptotic rates of miR-205-5p overexpressed EC109 and EC520 cells were significantly higher than those of control cells (Fig. 2c). Meanwhile, miR-205-5p knockdown in normal endometrial stromal cells showed similar functional phenotypes as endometriosis-derived endometrial stromal cells (Additional file 5: Figure S3). More importantly, western blot analysis showed that miR-205-5p overexpression significantly upregulated Bax and E-Cad protein expression but downregulated Bcl-2 and Vimentin protein expression (Additional file 6: Figure S4). Taken together, these results showed that miR-205-5p suppressed migration and invasion but promoted apoptosis of endometriosis-derived endometrial stromal cells.

### miR-205-5p suppressed endometriosis progression in vivo

**Fig. 3:**
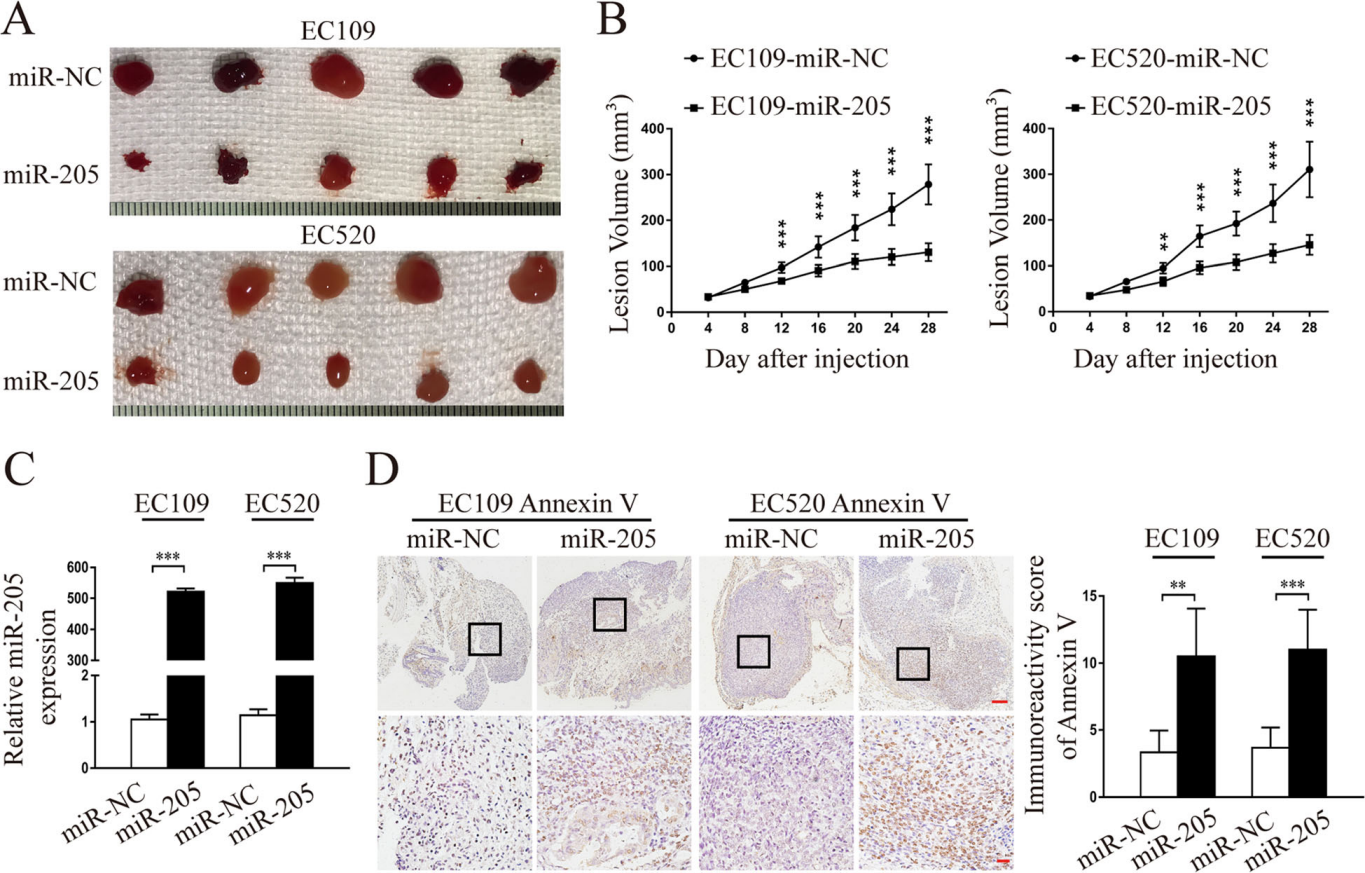
miR-205-5p suppressed the formation of endometrial-like lesions derived from endometrial stromal cells in vivo*.* **a** Representative micrographs of dissected lesions from nude mice (*n* = 6, per group) after 28 days of implantation. **b** The endometrial-like lesion growth curve. The lesion sizes were measured at 4-day intervals. **c** miR-205-5p levels in dissected lesions formed by EC109 and EC520 stably transfected with miR-205-5p or miR-NC were analysed by qRT-PCR. **d** Representative micrographs of Annexin V staining in dissected lesions. Scale bars: upper panel, 100 μm; lower panel, 20 μm. miR-205, miR-205-5p. Error bars represent the mean ± SD of three independent experiments. **P < 0.01; ***P < 0.001

To better evaluate the biologic function of miR-205-5p in vivo, we performed an in vivo endometriosis study by inoculating EC109 and EC520 cells stably expressing miR-205-5p or NC into nude mice. The miR-205-5p-overexpressed EC109 and EC520 cells formed significantly smaller endometrial-like lesions (Fig. 3a) and markedly slowed lesion xenograft growth compared with NC (Fig. 3b). qRT-PCR analysis confirmed that miR-205-5p levels were increased in miR-205-5p-xenografted lesions compared with NC (Fig. 3c). In addition, Annexin V staining showed that lesion xenografts of miR-205-5p-overexpressed EC109 and EC520 cells had more apoptotic cells than NC (Fig. 3d). Furthermore, miR-205-5p overexpression significantly increased the secretion of associated inflammatory cytokines in the pathogenesis of endometriosis, such as interleukin-1 beta (IL-1β), interleukin-6 (IL-6), soluble tumour necrosis factor α receptors 1 and 2 (sTNFR-1 and 2), and high-sensitivity C-reactive protein (hs-CRP) (Additional file 7: Figure S5). These results demonstrated that miR-205-5p could exert a significant inhibitory effect on endometriosis of endometrial stromal cells in vivo*.*

### ANGPT2 is the direct target of miR-205-5p

**Fig. 4:**
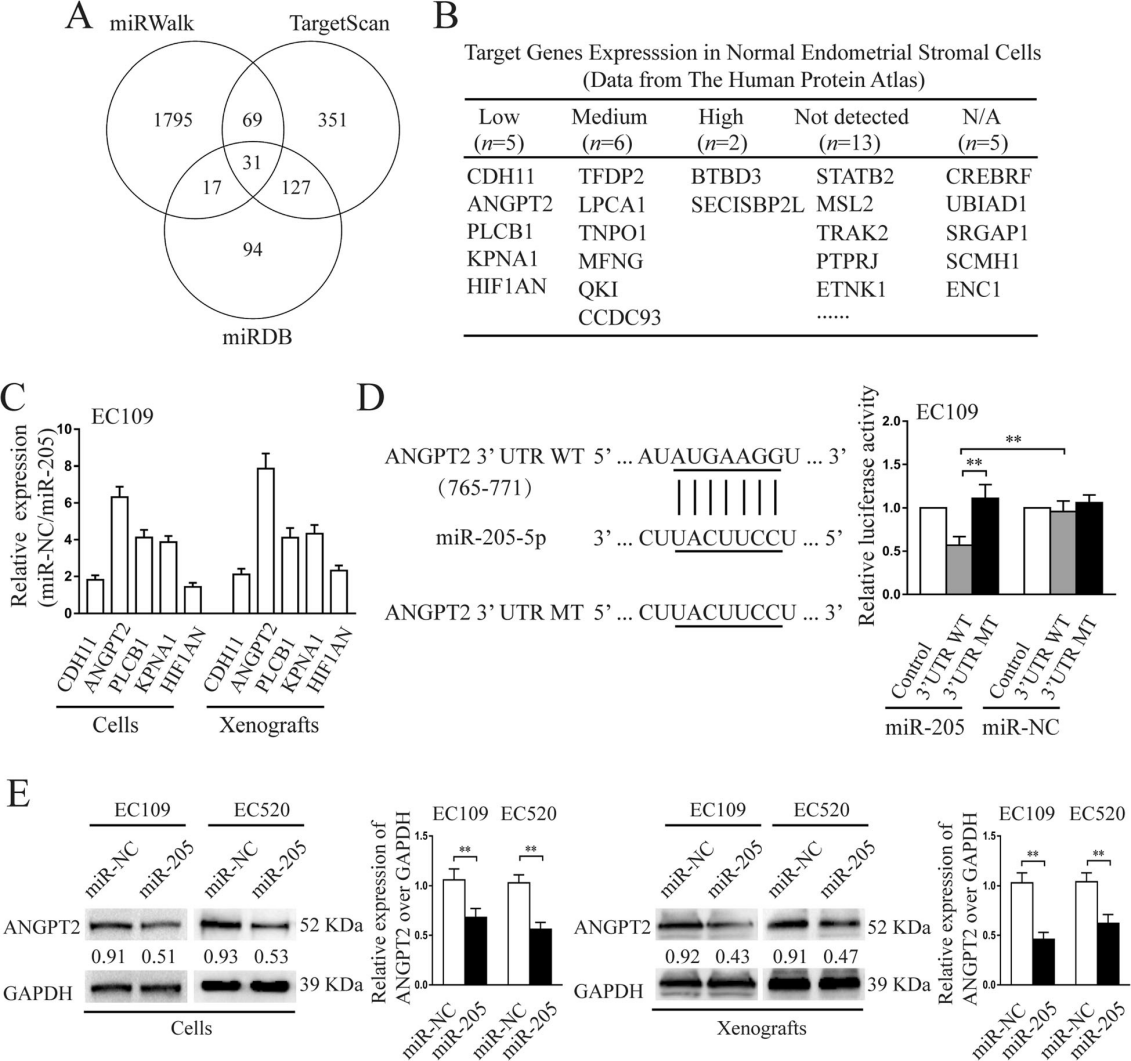
miR-205-5p directly inhibited ANGPT2 expression via its 3′-UTR. **a** Overlap of three miRNA target bioinformatic prediction algorithms. **b** Expression of predicted targets in normal endometrial stromal cells. Data from The Human Protein Atlas. **c** The RNA levels of CDH11, ANGPT2, PLCB1, KPNA1, and HIF1AN in cells and xenografts associated with miR-205-5p-overexpressed EC109 cells were analysed by qRT-PCR. **d** RNA sequence alignment between miR-205-5p and the 3′-UTR of ANGPT2 (left), and the effect of miR-NC and miR-205-5p on the activity of the luciferase reporter containing either wild type (WT) or mutant type (MT) in EC109 were tested by dual-luciferase reporter assay (right). **e** The ANGPT2 protein levels in EC109 and EC520 cells transfected by lentivectors and in the xenografted lesions established by these ectopic endometrial stromal cells were detected by the western blot analysis. miR-205, miR-205-5p. Error bars represent the mean ± SD of three independent experiments. **P < 0.01

Multiple algorithms (miRWalk, TargetScan, miRDB) were used to identify the candidate targets of miR-205-5p. As a result, 31 target genes were predicted to be regulated by miR-205-5p (Fig. 4a). Among these candidates, CDH11, ANGPT2, PLCB1, KPNA1, and HIF1AN lowly expressed in normal endometrial stromal cells confirmed by The Human Protein Atlas (THPA) database and were chosen for the next study (Fig. 4b). To further verify this prediction, a qRT-PCR analysis was performed and the results showed that the CDH11, ANGPT2, PLCB1, KPNA1, and HIF1AN expression in cells and xenografts associated with miR-205-5p-overexpressed EC109 and EC520 cells were all downregulated at mRNA levels (Fig. 4c; Additional file 8: Figure S6A). Among them, ANGPT2 was the most significantly downregulated gene.

To examine miR-205-5p regulation of the putative target ANGPT2, the predicted miR-205-5p binding site in the 3′-UTR of ANGPT2 (wild type) or the mutated sequence (mutant type) was cloned into luciferase reporter plasmids and assessed for their response to miR-205-5p in EC109 and EC520 cells. The results showed that the expression of the reporter gene followed by a wild-type 3′-UTR of ANGPT2 was significantly reduced by the co-transfected miR-205-5p mimic, whereas reporter gene expression had no change if followed by the 3′-UTR of the ANGPT2 gene with a mutated putative target site of miR-205-5p (Fig. 4d; Additional file 8: Figure S6B). Thus, we concluded that ANGPT2 is a direct target of miR-205-5p.

To confirm the downregulation of miR-205-5p on ANGPT2 protein levels, western blot analysis was performed to detect the ANGPT2 protein levels in EC109 and EC520 cells transfected by lentivectors and in the xenografted lesions established by these ectopic endometrial stromal cells. ANGPT2 protein levels were markedly downregulated in miR-205-5p-overexpressed EC109 and EC520 cells (Fig. 4e). The similar results were observed in the corresponding xenografted lesions formed by miR-205-5p-overexpressed EC109 and EC520 cells (Fig. 4e). Overall, miR-205-5p negatively regulated ANGPT2 expression in vitro and in vivo.

### ANGPT2 was a critical downstream mediator of miR-205-5p-suppressive effects in endometriosis

**Fig. 5:**
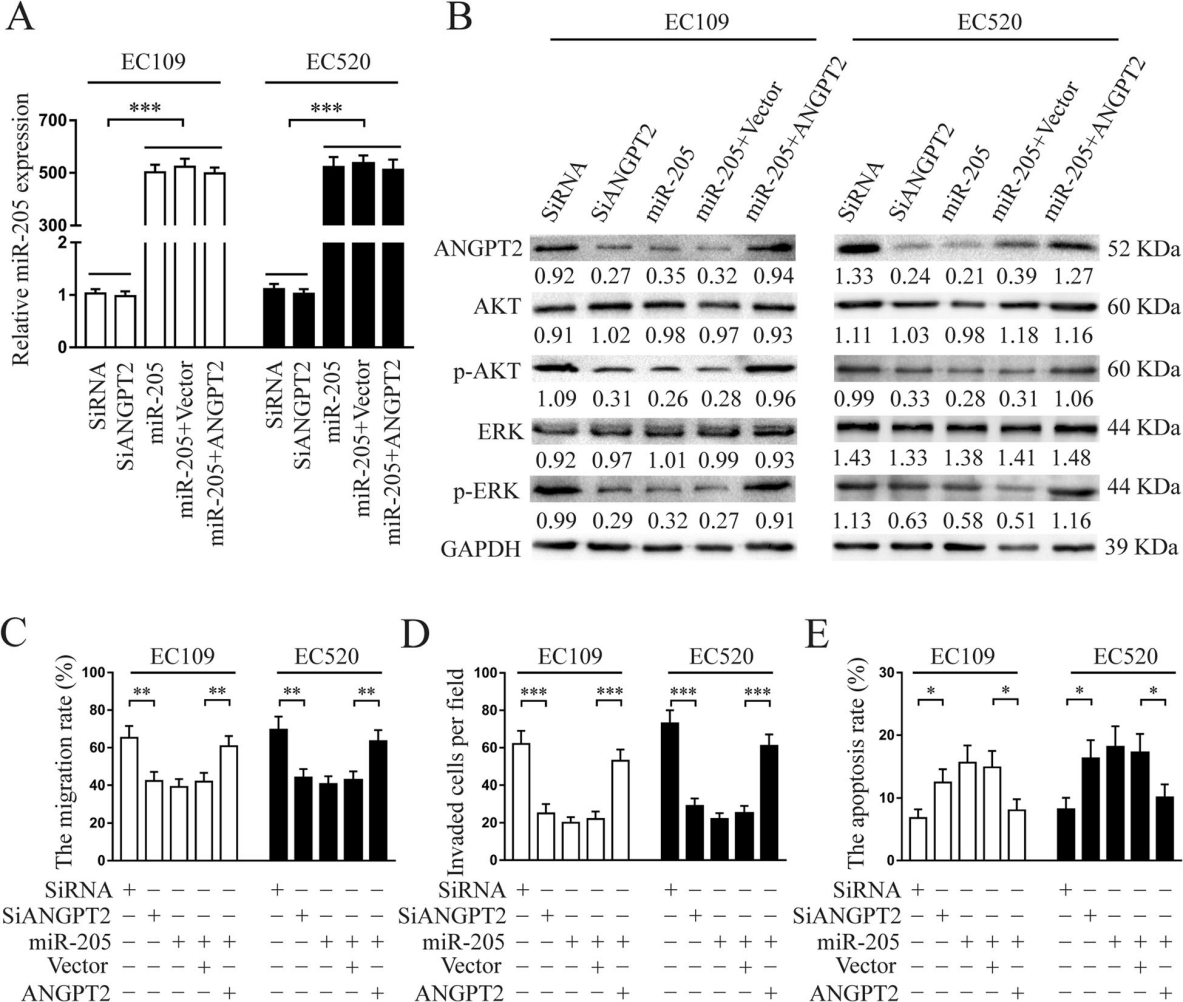
The re-expression of ANGPT2 could rescue the suppressive effects of miR-205-5p in endometriosis. **a** The levels of miR-205-5p in cells with indicated treatment were analysed by qRT-PCR. **b** The expression of ANGPT2 and AKT/ERK pathway-associated protein in Lenti-miR-205-5p-treated cells transfected with plasmid-ANGPT2 or vector control and SiANGPT2- or SiRNA-treated cells were detected by western blot analysis. **c**–**e** Re-expression of ANGPT2 rescued, whereas knockdown of ANGPT2 simulated the functional effects induced by miR-205-5p overexpression through wound healing, Transwell invasion and apoptosis assay. miR-205, miR-205-5p. These experiments were conducted after the indicated treatment for 48 h. Error bars represent the mean ± SD of three independent experiments. *P < 0.05; **P < 0.01; ***P < 0.001

To explore whether the role of miR-205-5p in endometriosis was mediated through suppressing ANGPT2, plasmids expressing ANGPT2 without 3′-UTR and specific small-interfering RNA-SiANGPT2 were conducted. Previous studies suggested that AKT and ERK pathways were closely related to endometriosis progression [20–22], prompting us to determine whether the AKT and ERK pathways could be inhibited by miR-205-5p. Therefore, the expression of miR-205-5p, ANGPT2 protein, and AKT/ERK pathway-associated protein in Lenti-miR-205-5p-treated cells transfected with plasmid-ANGPT2 and SiANGPT2-treated cells for 48 h were respectively detected by qRT-PCR and western blot (Fig. 5a, b). As the results showed, re-expression of ANGPT2 rescued, whereas knockdown of ANGPT2 simulated, the suppression of ANGPT2 and AKT/ERK pathway activation mediated by miR-205-5p.

Further in vitro studies confirmed that re-expression of ANGPT2 dramatically attenuated the effects induced by miR-205-5p. Meanwhile, the knockdown of ANGPT2 generated similar functional phenotypes associated with miR-205-5p overexpression in ectopic endometrial stromal cells, including suppressing the abilities of migration and invasion (Fig. 5c, d; Additional file 9: Figure S7A & B), as well as promoting cellular apoptosis (Fig. 5e; Additional file 9: Figure S7C). Collectively, these results indicated that ANGPT2 was a critical downstream mediator of miR-205-5p suppressive effects in endometriosis.

### Clinical associations of miR-205-5p with ANGPT2 expression in human endometriosis tissues

**Fig. 6:**
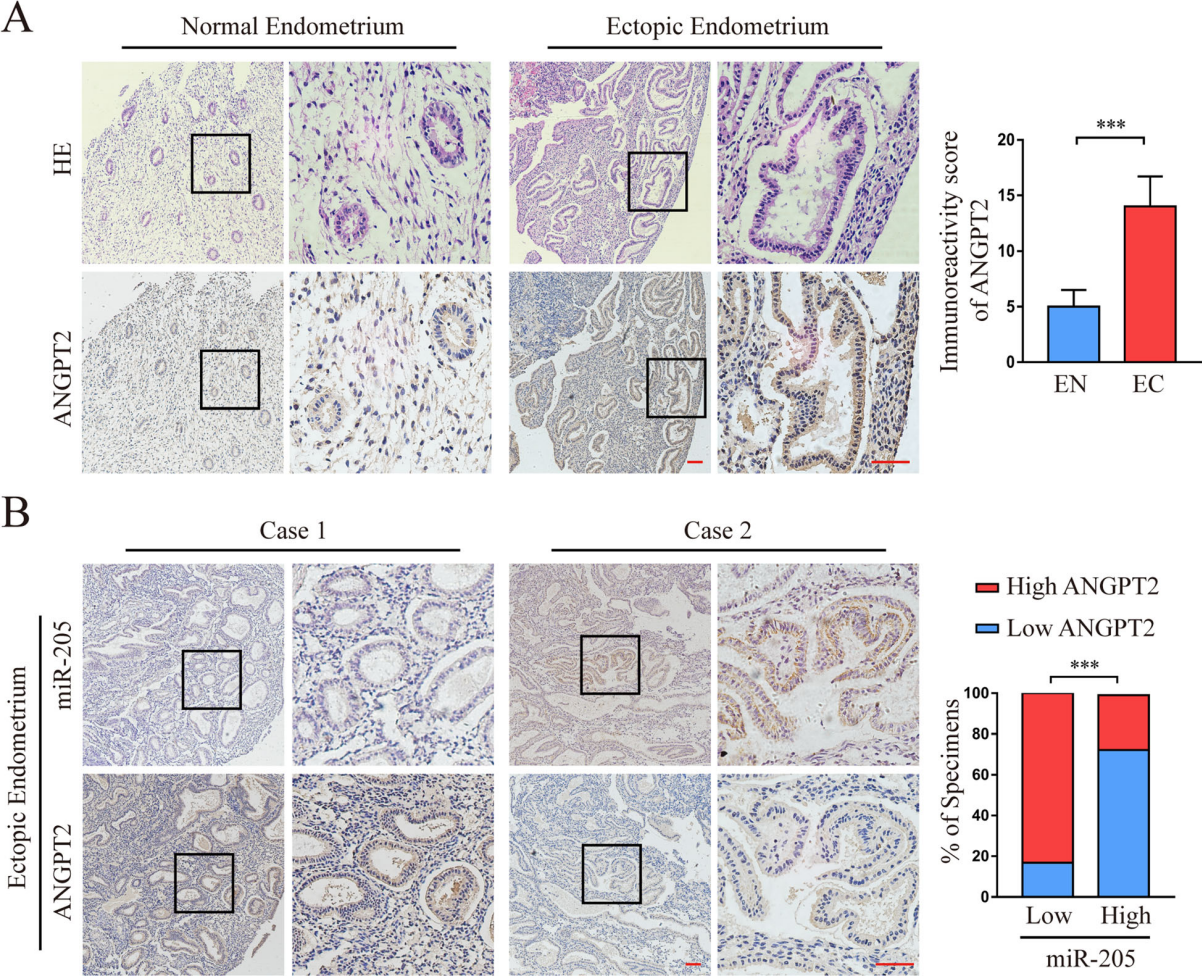
Association between miR-205-5p and ANGPT2 expression in clinical tissues. **a** Immunoreactivity score of ANGPT2 was analysed between the EN (*n* = 23) and EC (*n* = 68) clinical specimens. **b** Representative images and the percentages of tissues with high and low ANGPT2 expression with low or high levels of miR-205-5p in EC clinical specimens. Scale bars, 100 μm. EN, normal endometrium; EC, ectopic endometrium. miR-205, miR-205-5p. Error bars represent the mean ± SD of three independent experiments. ***P < 0.001

We first used the antibody that specifically recognised ANGPT2 to examine the expression pattern in the aforementioned 68 EN and 23 EC clinical specimens. As shown in Fig. 6a, the expression of ANGPT2 protein in EC tissues was significantly higher than that in EN tissues. We further investigated whether there was an association between the expression of ANGPT2 and miR-205-5p in human endometriosis tissues. The results showed that the expression of ANGPT2 protein was negatively correlated with miR-205-5p levels in the EC tissues (Fig. 6b; Additional file 1: Table S4), suggesting that miR-205-5p suppressed ANGPT2 expression in clinical endometriosis progression.

Moreover, we analysed the relationship between clinicopathological characteristics and miR-205 expression in patients with endometriosis. The dysmenorrhoea score, CA125 level, and endometriosis score were dramatically correlated with downregulation of miR-205-5p and upregulation of ANGPT2 (Table 1). More importantly, multivariate analysis confirmed that downregulation of miR-205-5p and upregulation of ANGPT2 score were also markedly correlated with endometriosis score (Table 2). Taken together, these data indicated that miR-205-5p and ANGPT2 were promising biomarkers for reflecting the severity of endometriosis.

**Table 1:** Correlation between the clinicopathological factors and expression of miR-205-5p and ANGPT2 in endometriosis

	miR-205-5p expression			ANGPT2 expression		
	High	Low	*P*	High	Low	*P*
Patients (*n*)	19	49		45	23	
Age (years)	31.26 ± 5.98	31.98 ± 6.36	0.67	32.18 ± 6.43	31.00 ± 5.85	0.464
Haemoglobin (g/L)	113.89 ± 15.08	114.80 ± 18.00	0.85	113.98 ± 18.07	115.65 ± 15.44	0.706
Endometrial phase (%)						
Proliferative phase	78.95%	83.67%		82.22%	82.61%	
Secretive phase	21.05%	16.33%	0.917	17.78%	17.39%	0.97
Dysmenorrhoea pain score (%)						
Less than 4 points	57.89%	30.61%		28.89%	56.52%	
More than 4 points	42.11%	69.39%	0.038	71.11%	43.48%	< 0.001
Chronic pelvic pain score (%)						
Less than 4 points	42.11%	36.69%		31.11%	47.83%	
More than 4 points	57.89%	65.31%	0.57	68.89%	52.17%	0.18
E2 (pmol/L)	369.58 ± 355.63	296.55 ± 301.26	0.245	276.76 ± 279.00	395.61 ± 373.30	0.054
CA-125 (kU/L)	34.31 ± 21.27	109.55 ± 140.91	0.001	116.67 ± 144.86	33.47 ± 21.40	< 0.001
Endometriosis score (%)						
Less than 16 points	68.42%	38.78%		35.56%	69.57%	
More than 16 points	31.58%	61.22%	0.028	64.44%	30.43%	0.008

**Table 2:** Summary of univariate and multivariate Cox regression analysis of the severity of endometriosis

	Univariate analysis			Multivariate analysis		
	SE	B	*P*	SE	B	*P*
Age (years)	0.132	− 0.517	< 0.001	0.003	− 0.031	< 0.001
Haemoglobin (g/L)	0.025	− 0.001	0.971	0.059	0.001	0.552
Endometrial phase	1.034	−0.382	0.712	3.012	0.096	0.139
Dysmenorrhoea pain score	0.646	2.066	0.001	1.669	0.217	< 0.001
Chronic pelvic pain score	0.644	2.128	0.001	1.577	0.103	0.051
E2 (pmol/L)	0.017	0.011	0.313	0.12	0.001	0.121
CA-125 (kU/L)	0.054	0.157	0.004	0.08	0.001	0.001
miR-205-5p expression	0.573	2.228	< 0.001	1.688	1.628	< 0.001
ANGPT2 expression	0.67	−2.56	< 0.001	1.581	− 0.203	0.008

## Discussion

A clinical common misinterpretation of endometriosis-induced pain as menstrual-related abdominal pain, as well as the invasive nature of laparoscopy and lack of a laboratory biomarker for the disease, results in an average latency of 7 to 11 years from the onset of symptoms to definitive diagnosis [23–25]. In 2004, Ballweg reported an increase of endometriosis-like symptoms in girls before the age of 15 years as well as those with earlier menarche [26], indicating the extreme necessity to identify novel and effective biomarkers that can screen adolescent girls as early as they display symptoms of endometriosis. The identification of biomarkers will contribute to developing personalised diagnostic and therapeutic strategies for patients with confirmatory diagnosis. Recently, miRNAs have been recognised to be promising biomarkers because of their tissue-specific expression profiles and minimally invasive diagnostic means [27–29]. In our study, miR-205-5p was identified as a novel pathologic suppressor of endometriosis using tissue microarray analysis. We further confirmed that miR-205-5p could directly target angiopoietin-2 (ANGPT2) by binding to its 3′-UTR and be involved in the procession of endometriosis via regulating the ANGPT2 pathway. More importantly, our clinical data showed that both miR-205-5p and ANGPT2 are valuable factors for reflecting the severity of endometriosis. These results provided us with enough reason to further explore the roles of the miR-205-5p-ANGPT2 axis in the progression of endometriosis.

Profiles of miRNA expression in normal endometrium exhibit dynamic changes across the menstrual cycle [30], where their implication into physiologic systems can be used to identify pathologic phenotypes. Endometriosis can be typically categorised into two types: asymptomatic and symptomatic, depending on the arrival location of endometrial-like tissues [31–33]. As expected, the miRNA profiles of ectopic endometrium are more representable than those of eutopic endometrium, which provides more useful pathophysiologic fingerprints to confirm a diagnosis of symptomatic endometriosis. Here, we found that miR-205-5p levels in both tissues and serum from ectopic endometrium patients were significant downregulation compared with those from normal endometrium patients. Moreover, our study data confirmed the suppressive effect of miR-205-5p through primary ectopic endometrial stromal cell migration, invasion, and apoptosis assay in vitro, along with endometrial-like xenograft growth and apoptosis in vivo. Although endometriosis cells can hardly transform into cancer cells, the cancerous behaviour, such as invasion into adjacent organs and spreading to distant organs, frequently appears in endometriosis [34, 35]. Interestingly, previous studies supported our data that the overexpression of miR-205-5p in multiple cancer types induced similar in vitro and in vivo phenotypes to our study [36–38], indicating that miR-205-5p could be an ideal therapeutic target that contributes to suppressing the cancerous behaviour.

To further explore the molecular mechanisms underlying the involvement of miR-205-5p in human endometriosis, we combined three typical miRNA prediction algorithms and the THPA database to identify the potential targets of miR-205-5p. THPA is an online platform for simultaneously identifying and quantifying protein of potential targets in both human physiologic and pathologic tissues [39]. Compared with the conventional qRT-PCR analysis for targets of miRNAs, THPA can be used as a better identification and quantification tool for primarily screening target protein due to its large and accurate data in human tissues. Then, we integrated the prediction results of bioinformatic methods and luciferase reporter assay. Consequently, ANGPT2 was identified as a novel direct target of miR-205-5p. Various genes have been identified as the direct targets of miR-205-5p. For example, miR-205-5p directly targeted ERBB2 and p63, leading to resistance to standard therapy for Her2-positive breast cancer [40]. A lipid metabolism-related gene called acetyl-CoA carboxylase β (ACACβ) was targeted by miR-205-5p in hepatic lipid metabolism [41]. A recent study by Di Carlo et al. found that angiopoietin-2 (ANGPT2) was gradually downregulated in normal endometrium, eutopic endometrium, and ectopic endometrium using immunohistochemical staining [42]. However, up to now, the underlying mechanism for ANGPT2 affecting the pathogenesis of endometriosis is still unclear. ANGPT2, a well-recognised vascular destabilising factor, is a biomarker of poor outcome in many human diseases [43, 44]. Our data here suggested that overexpression of miR-205-5p could cause the significant downregulation of ANGPT2 at both mRNA and protein levels in ectopic endometrial stromal cells and xenografted lesions. Meanwhile, the re-expression and knockdown of ANGPT2 could respectively rescue and simulate the effects induced by miR-205-5p. Moreover, activation of the AKT and ERK pathways involved in endometriosis progression was responsible for the downregulation of miR-205-5p and upregulation of ANGPT2.

Although decades of research have gone into developing a set of diagnostic biomarkers for evaluating the severity of endometriosis, the effective biomarkers for differentiating different types and degrees of endometriosis are still lacking. For example, CA-125 is a clinical biomarker for the prediction of endometriosis severity [45]. However, the correlation of CA-125 levels with disease progression is not high [46]. Several other schemes that are being implemented to detect inflammatory biomarkers such as IL-8 and IL-6 are still unsatisfactory [47, 48]. Based on the widely accepted theory on the retrograde movement of sloughed menstruation [49], the menstrual endometrial cells are the source of ectopic endometriotic foci. Therefore, using the direct source of the disease, including peripheral blood or even urine, is perhaps reasonable and logical in our quest to identify biomarkers for endometriosis. In our study, ISH and IHC analyses in human ectopic endometrium serial sections also showed an adverse relationship between miR-205-5p and ANGPT2. More importantly, we showed that clinical endometriosis severity scores are also closely correlated with miR-205-5p and ANGPT2 expression, and predicting endometriosis severity was also determined according to the multivariate regression model.

## Conclusion

Taken together, our data provided evidence that miR-205-5p may function as an ectopic endometriotic suppressor when evaluating the disease severity in human endometriosis. Decreased miR-205-5p may contribute to apoptosis reduction and promoting migration and invasion by regulating the ANGPT2-AKT/ERK pathway. The newly identified miR-205-5p-ANGPT2-AKT/ERK signalling axis illustrated a critical molecular mechanism of endometriosis progression and provided a novel diagnostic and therapeutic target for endometriosis treatment.

## Additional files

Additional file 1: **Table S1.** Descriptive characteristics of patients with endometriosis. **Table S2.** Detailed primer sequences in the study. **Table S3.** The antibodies used in western blot. **Table S4.** Expression of miR-205-5p and ANGPT2 in endometriosis, related to Figure 6. (DOCX 20 kb)

Additional file 2: Supplemental materials and methods. (DOCX 16 kb)

Additional file 3: **Figure S1.** Identification of endometrial stromal cells, related to Fig. 1. a. Representative fluorescent images of vimentin and cytokeratin expression in EC109, EC520, EN211, and EN307 cells. b. Representative morphological changes of EC109, EC520, EN211, and EN307 cells induced with 10^-8^ M E2 + 10^-7^ M MPA for 14 d. c. The PRL protein levels in supernatant of E2+MPA-induced cells was detected by ELISA. Scale bar, 20 μm. Error bars represent the mean ± SD of three independent experiments. ***, *P*<0.001. (TIF 4359 kb)

Additional file 4: **Figure 2.** Primary endometrial stromal cells stably expressed miR-205-5p after lentivectors transfection, related to Fig. 2. a. Representative fluorescent images of EC109 and EC520 after lentivectors transfection. Scale bar, 20 μm. b. miR-205-5p levels in EC109 and EC520 stably transfected with miR-205-5p (miR-205) or negative control (miR-NC) lentivectors were detected by qRT-PCR. c. miR-205-5p levels in EN, EC109 and EC520 were detected by qRT-PCR. d. miR-205-5p levels in EC109 and EC520 transiently transfected with miR-205-5p inhibitors (anti-205) or negative control (miR-NC) were detected by qRT-PCR. EN, normal endometrium. miR-205, miR-205-5p. anti-205, anti-205-5p. Error bars represent the mean ± SD of three independent experiments. ***, *P*<0.001. (TIF 4478 kb)

Additional file 5: **Figure S3.** miR-205-5p knockdown promoted migration and invasion but suppressed apoptosis of EN211 and EN307 in vitro, related to Fig. 2. a. miR-205-5p levels in EN211 and EN307 stably transfected with anti-205-5p (anti-205) or negative control (anti-NC) lentivectors were detected by qRT-PCR. b. Representative micrographs of wound healing assay in miR-205-5p-knockdown EN211 and EN307 compared with NC were shown. Images were acquired at 0 (white dotted line) and 48 h (black dotted line). Average migration rate per field was calculated. Scale bar, 20 μm. c. Representative micrographs of Transwell invasion assay in miR-205-5p- knockdown EN211 and EN307 compared with NC were shown. Average invasive cells per field were calculated. Scale bar, 50 μm. d. Representative micrographs of apoptosis assay in miR-205-5p-knockdown EN211 and EN307 compared with NC were shown. Average apoptosis rate per time was analysed. anti-205, anti-miR-205-5p. Error bars represent the mean ± SD of three independent experiments. **, *P*<0.01; ***, *P*<0.001. (TIF 4383 kb)

Additional file 6 **Figure S4.** The protein levels associated with migration, invasion and apoptosis in EC109 and EC520 cells transfected by lentivectors were detected by the western blot analysis, related to Fig. 2. miR-205, miR-205-5p. (TIF 937 kb)

Additional file 7: **Figure S5.** The protein levels of interleukin-1 beta (IL-1 β), interleukin-6 (IL-6), soluble tumor necrosis factor α receptors 1 and 2 (sTNFR-1 and sTNFR-2), and high-sensitivity C-reactive protein (hs-CRP) in peripheral blood from animal model of endometriosis were detected by ELISA, related to Fig. 3. miR-205, miR-205-5p. Error bars represent the mean ± SD of three independent experiments. ***, *P*<0.001. (TIF 509 kb)

Additional file 8: **Figure S6.** miR-205-5p directly inhibited ANGPT2 expression via its 3’-UTR, related to Fig. 4. a. The RNA levels of CDH11, ANGPT2, PLCB1, KPNA1 and HIF1AN in cells and xenografts associated with miR-205-5p-overexpressed EC520 cells were analysed by qRT-PCR. b. The effect of miR-NC and miR-205-5p on the activity of the luciferase reporter containing either wild type (WT) or mutant type (MT) in EC520 were tested by dual-luciferase reporter assay. miR-205, miR-205-5p. Error bars represent the mean ± SD of three independent experiments. **, *P*<0.01. (TIF 483 kb)

Additional file 9: **Figure S7.** The re-expression of ANGPT2 could rescue the suppressive effects of miR-205-5p in endometriosis, related to Fig. 5. a. Representative images of wound healing assay in EC109 and EC520 treated as indicated were shown. Images were acquired at 0 (white dotted line) and 48 h (black dotted line). Scale bar, 20 μm. b. Representative images of Transwell invasion assay in EC109 and EC520 treated as indicated were shown. Scale bar, 50 μm. c. Representative images of apoptosis assay in EC109 and EC520 treated as indicated were shown. (TIF 9701 kb)

## Data Availability

The raw data of miRNA microarray have been submitted to GEO (accession number: GSE124010).

## Abbreviations

ANGPT2: Angiopoietin-2
UTRs: Untranslated regions
PBS: Phosphate-buffered saline
qRT-PCR: Quantitative reverse transcriptase polymerase chain reaction
GAPDH: Glyceraldehyde-3-phosphate dehydrogenase
IHC: Immunohistochemistry
ISH: In situ hybridization
SD: Standard deviation
EN: Normal endometrium
EC: Ectopic endometrium
THPA: The Human Protein Atlas
WT: Wild type
MT: Mutant type
E2: Estradiol
MPA: Medroxyprogesterone acetate
PRL: Prolactin
IL-1β: Interleukin-1 beta
IL-6: Interleukin-6
sTNFR-1: Soluble tumour necrosis factor α receptor 1
sTNFR-2: Soluble tumour necrosis factor α receptor 2
hs-CRP: High-sensitivity C-reactive protein
