# Correction to: miR-205-5p inhibits human endometriosis progression by targeting ANGPT2 in endometrial stromal cells

**DOI:** 10.1186/s13287-020-01686-8

**Published:** 2020-05-20

**Authors:** Chen-Fei Zhou, Min-Juan Liu, Wei Wang, Sha Wu, Yu-Xin Huang, Guo-Bin Chen, Li-Min Liu, Dong-Xian Peng, Xue-Feng Wang, Xu-Zi Cai, Xiao-Xuan Li, Wan-Qin Feng, Ying Ma

**Affiliations:** 1 Department of Obstetrics and Gynecology, The First Affiliated Hospital of Guangzhou Medical University, Guangzhou, 510120 China; 2 Department of Obstetrics and Gynecology, Zhujiang Hospital of Southern Medical University, No.253, Middle Gongyeda Road, Haizhu District, Guangzhou, 510280 China; 3 Department of Immunology/Guangdong Provincial Key Laboratory of Proteomics, School of Basic Medical Sciences, Southern Medical University, Guangzhou, 510515 China; 4 Department of Obstetrics and Gynecology, Shenzhen Maternal and Child Healthcare Hospital of Southern Medical University, Shenzhen, 518028 China

**Correction to: Stem Cell Res Ther (2019) 10:287**

**https://doi.org/10.1186/s13287-019-1388-5**

The original article [1] contains an error in Fig. 1 whereby in sub-panel A of Fig. 1, the blue and red thresholds were mistakenly reversed. The correct version of sub-panel A in Fig. 1 can be viewed ahead.

**Fig. 1:**
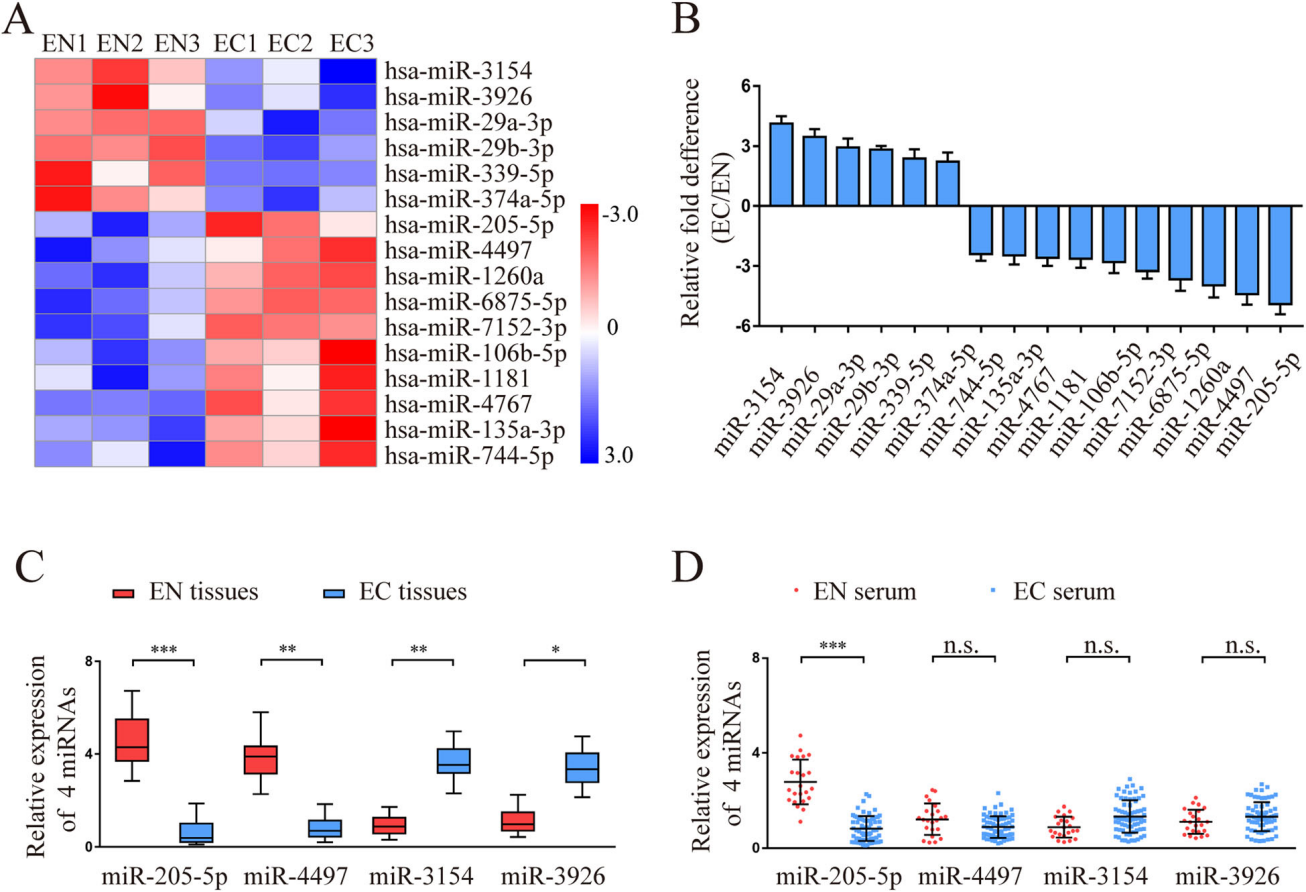
Identification of miR-205-5p as a negatively pathologic miRNA in endometriosis. **a** The different miRNA expression profiles between the EN (*n* = 3) and EC (*n* = 3) groups were analysed by miRNA microarray. The heatmap diagram shows that the representative miRNAs were significantly associated with endometriosis. EN1, EN2, and EN3 indicate 3 normal endometria; EC1, EC2, and EC3 indicate 3 ectopic endometria. **b** The levels of 16 differentially expressed miRNAs in the endometrium used for microarray analysis were analysed by qRT-PCR. **c**, **d** The levels of miR-205-5p, miR-4497, miR-3154, and miR-3926 were validated by qRT-PCR in additional tissues and serum from the EN (*n* = 23) and EC (*n* = 68) groups. EN, normal endometrium; EC, ectopic endometrium. Error bars represent the mean ± SD of 3 independent experiments. n.s., not significant; *P < 0.05; **P < 0.01; ***P < 0.001
