# Supplementary material for: miR-205-5p inhibits human endometriosis progression by targeting ANGPT2 in endometrial stromal cells
Source: Stem Cell Res Ther. 2019 Sep 23;10:287. doi: 10.1186/s13287-019-1388-5 (PMC6757391; doi:10.1186/s13287-019-1388-5)
Supplement: Supplementary file 1 — Table S1. Descriptive characteristics of patients with endometriosis. Table S2. Detailed primer sequences in the study. Table S3. The antibodies used in western blot. Table S4. Expression of miR-205-5p and ANGPT2 in endometriosis, related to Figure 6. (DOCX 20 kb) [file 13287_2019_1388_MOESM1_ESM.docx]

**Additional file 1**

**Table S1. Descriptive characteristics of patients with endometriosis**

|  | Control | Endometriosis | *P* |
| --- | --- | --- | --- |
| Patients(n) | 26 | 71 |  |
| Age (years) | 46.69±4.15 | 31.84±6.28 | <0.005 |
| Haemoglobin (g/l) | 106.31±18.57 | 114.42±16.78 | 0.043 |
| Endometrial phase (%) |  |  |  |
| Proliferative phase | 76.92% | 81.69% |  |
| secretive phase | 23.08% | 18.31% 0.600 | |
| Dysmenorrhoea pain score (%) |  |  | |
| less than 4 points | 80.77% | 28.17% | |
| more than 4 points | 19.23% | 71.83% <0.001 | |
| Chronic pelvic pain score（%）  less than 4 points | 84.62% | 36.62% | |
| more than 4 points | 15.38% | 63.38% | <0.001 |
| E2 (pmol/L) | 124.15±2.09 | 317.96±37.16 | <0.001 |
| CA-125 (kU/L) | 17.14±1.36 | 85.36±14.57 | <0.001 |

**Table S2. Detailed primer sequences in the study**

|  | Forward | Reverse |
| --- | --- | --- |
| CDH11 | GCCCCAAGTTACATCCACGA | ATGTCTTCCCTGGGAGAGGG |
| ANGPT2 | TTGGCCGCAGCCTATAACAA | CCGCTGTTTGGTTCAACAGG |
| PLCB1 | GGCACCTGCCAAAACAGAAG | CTGCAGCTTGGGCTTTTCAT |
| KPNA1 | CGTCTACCCACAGGCAACTT | ACCCCTTGACTCCAGGAAAAC |
| HIF1AN | GGATGAATCCCAGAGCCTGT | GTGCAGCGTGCAATACTAGC |
| GAPDH | CCATCAATGACCCCTTCATTGACC | GAAGGCCATGCCAGTGAGCTTCC |
| SiANGPT2 | GCAACGCUAUGUGCUUAAATT | UUUAAGCACAUAGCGUUGCTT |
| SiRNA | UUCUCCGAACGUGUCACGUTT | ACGUGACACGUUCGGAGAATT |

**Table S3. The antibodies used in western blot**

| Name | Company | Product Code |
| --- | --- | --- |
| anti-ANGPT2 | Abcam | ab56301 |
| anti-phospho-AKT | Cell Signaling Technology | 9271 |
| anti-AKT | Cell Signaling Technology | 9272 |
| anti-phospho-ERK1/2 | Cell Signaling Technology | 4370 |
| anti-ERK1/2 | Cell Signaling Technology | 4695 |
| Bax | Abcam | ab32503 |
| Bcl-2 | Abcam | ab182858 |
| E-cad | Abcam | ab15148 |
| Vimentin | Abcam | ab8069 |
| anti-GAPDH | Cell Signaling Technology | 2118 |
| anti-rabbit immunoglobulin-G | Abcam | ab6721 |

**Table S4. Expression of miR-205-5p and ANGPT2 in endometriosis, related to Figure 6.**

|  | miR-205-5p | ANGPT2 |
| --- | --- | --- |
| Low expression (n) | 49 | 23 |
| High expression (n) | 19 | 45 |
