# Supplementary material for: miR-205-5p inhibits human endometriosis progression by targeting ANGPT2 in endometrial stromal cells
Source: Stem Cell Res Ther. 2019 Sep 23;10:287. doi: 10.1186/s13287-019-1388-5 (PMC6757391; doi:10.1186/s13287-019-1388-5)
Supplement: Supplementary file 2 — Supplemental materials and methods. (DOCX 16 kb) [file 13287_2019_1388_MOESM2_ESM.docx]

**Supplemental materials and methods**

**Luciferase reporter assay**

The expression of miR-205-5p targeted gene was measured by using a dual-luciferase reporter assay in EC109 and EC520 cells. The putative miR-205-5p complementary site in the 3’-UTR of ANGPT2 or its mutant sequence was cloned into the pmiR-RB-REPORT vector (RiboBio Inc.). Then, pmiR-RB-REPORT-ANGPT2-3’UTR-WT or pmiR-RB-REPORT-ANGPT2-3’UTR-MT were co-transfected into EC109 and EC520 cells with miR-205-5p mimic or its negative control in 48-well plates, collected 48 h after transfection and analysed using a Dual-Luciferase Reporter Assay System (Promega). The Firefly luciferase signal was used for normalisation.

**ELISA assay**

PRL protein levels was detected in the culture supernatant of endometrial stromal cells induced with E2+MPA using a human PRL ELISA Kit (Thermo Fisher) according to the manufacturer’s protocol. Protein levels of interleukin-1 β, interleukin-6, soluble tumor necrosis factor α receptors 1 and 2, and high-sensitivity C-reactive protein in peripheral blood from [animal model of endometriosis](https://www.ncbi.nlm.nih.gov/pmc/articles/PMC4855629/#path4657-sec-0006title) by using respective mouse-specific ELISA Kit (Thermo Fisher) according to the manufacturer’s instructions.

**Wound healing assay**

Cells were seeded onto a 6-well dish to reach a density of 5 × 10^5^ cells/well. A scratch wound was generated using a sterile 10-µL pipet tip, and floating cells were removed by washing with PBS. Images of the scratches were taken using an inverted microscope at 100 × magnification at 0 and 48 h of incubation. The migration rate (%) was measured as a ratio of occupied area to the total area using Image Olympus IX71.

**Transwell invasion assay**

1 × 10^5^ cells in 200-µL DMEM medium without FBS were seeded on an upper layer membrane precoated with Matrigel (BD Biosciences) of Transwell (Corning). In the lower chamber, 600-µL DMEM with 10% FBS was added as a chemoattractant. After the cells that invaded to the bottom of the insert membrane were fixed with methanol, the insert was stained with Giemsa (Sigma) and we counted the cell numbers under a microscope in five random fields (× 200).

**Apoptosis assay**

Cell apoptosis was detected using Annexin V-FITC kit (BD Biosciences) and 7-AAD (Invitrogen) according to the manufacturer’s protocol.
