# Supplementary figures and images for: miR-205-5p inhibits human endometriosis progression by targeting ANGPT2 in endometrial stromal cells
Source: Stem Cell Res Ther. 2019 Sep 23;10:287. doi: 10.1186/s13287-019-1388-5 (PMC6757391; doi:10.1186/s13287-019-1388-5)

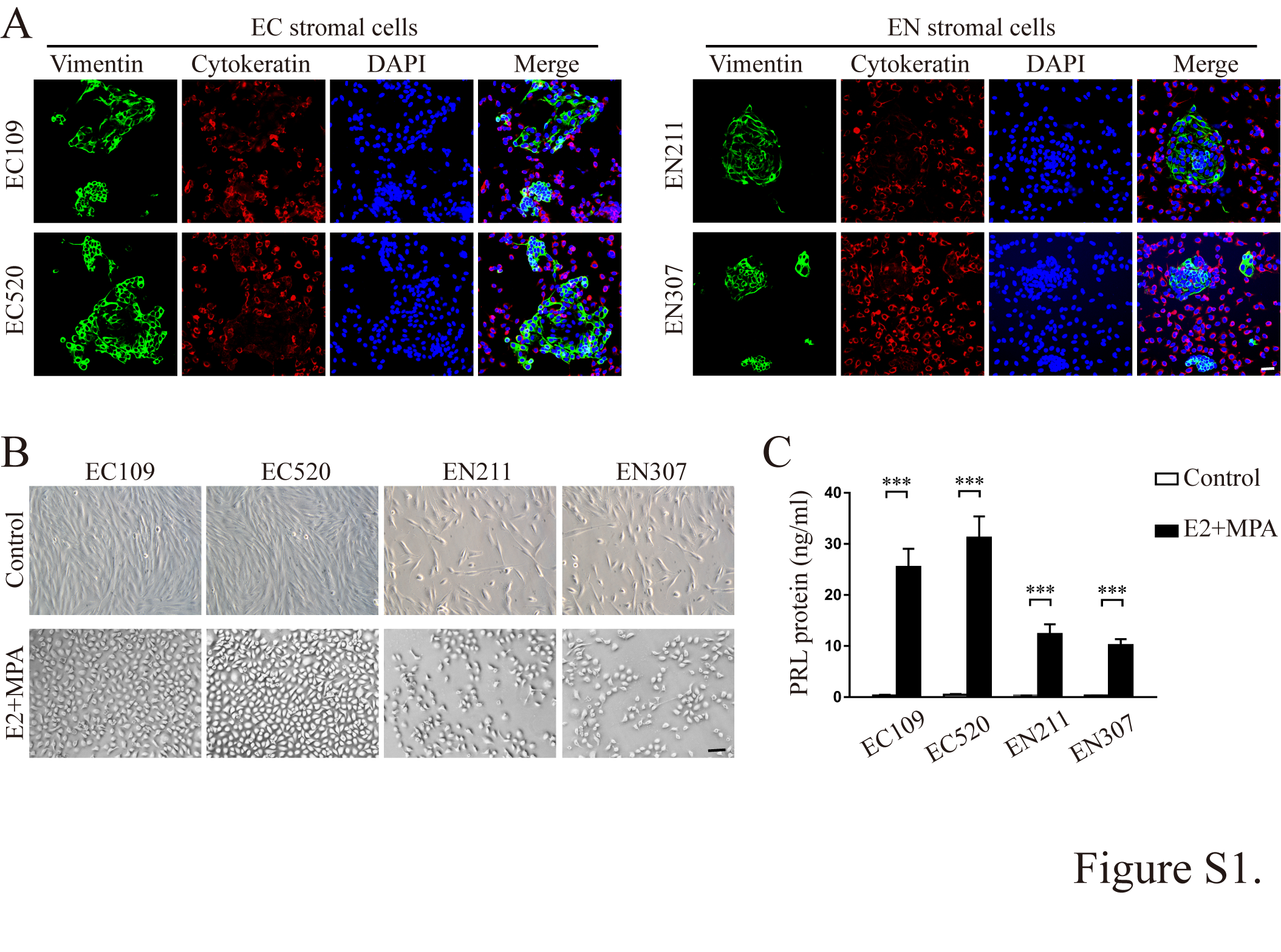

Supplement: Supplementary file 3 — Figure S1. Identification of endometrial stromal cells, related to Fig. 1. a. Representative fluorescent images of vimentin and cytokeratin expression in EC109, EC520, EN211, and EN307 cells. b. Representative morphological changes of EC109, EC520, EN211, and EN307 cells induced with 10-8 M E2 + 10-7 M MPA for 14 d. c. The PRL protein levels in supernatant of E2+MPA-induced cells was detected by ELISA. Scale bar, 20 μm. Error bars represent the mean ± SD of three independent experiments. ***, P<0.001. (TIF 4359 kb) [file 13287_2019_1388_MOESM3_ESM.tif]

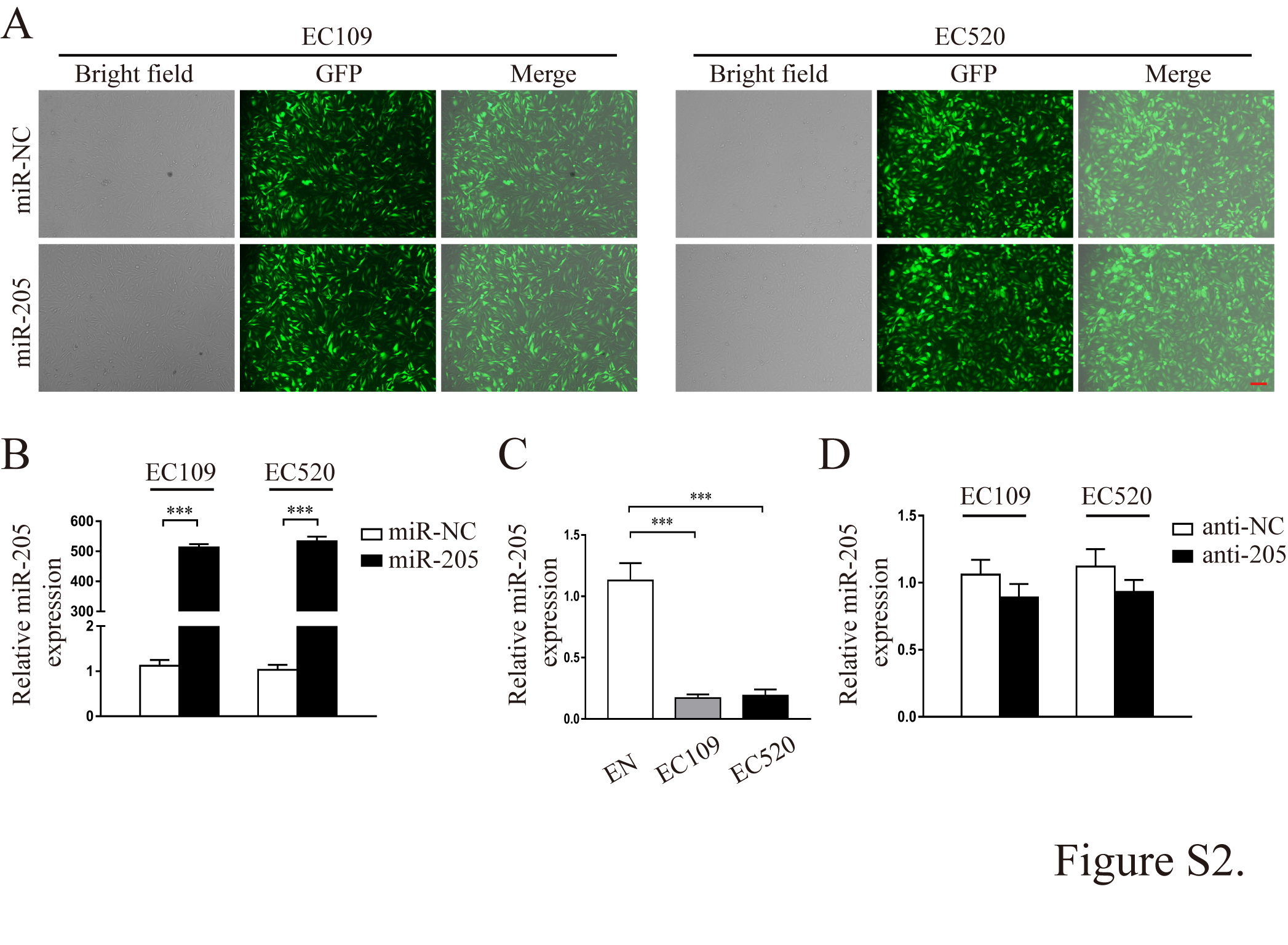

Supplement: Supplementary file 4 — Figure 2. Primary endometrial stromal cells stably expressed miR-205-5p after lentivectors transfection, related to Fig. 2. a. Representative fluorescent images of EC109 and EC520 after lentivectors transfection. Scale bar, 20 μm. b. miR-205-5p levels in EC109 and EC520 stably transfected with miR-205-5p (miR-205) or negative control (miR-NC) lentivectors were detected by qRT-PCR. c. miR-205-5p levels in EN, EC109 and EC520 were detected by qRT-PCR. d. miR-205-5p levels in EC109 and EC520 transiently transfected with miR-205-5p inhibitors (anti-205) or negative control (miR-NC) were detected by qRT-PCR. EN, normal endometrium. miR-205, miR-205-5p. anti-205, anti-205-5p. Error bars represent the mean ± SD of three independent experiments. ***, P<0.001. (TIF 4478 kb) [file 13287_2019_1388_MOESM4_ESM.tif]

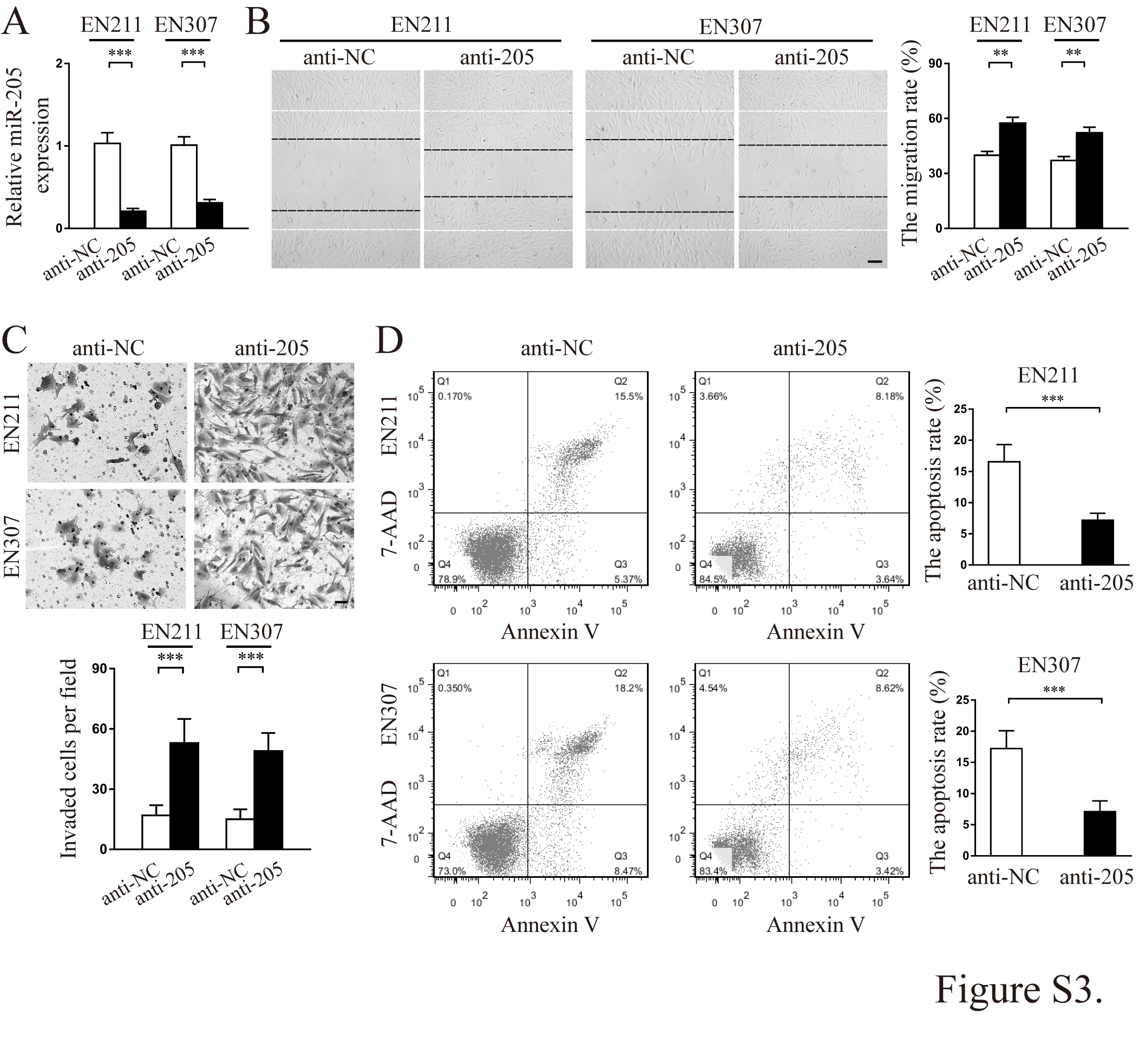

Supplement: Supplementary file 5 — Figure S3. miR-205-5p knockdown promoted migration and invasion but suppressed apoptosis of EN211 and EN307 in vitro, related to Fig. 2. a. miR-205-5p levels in EN211 and EN307 stably transfected with anti-205-5p (anti-205) or negative control (anti-NC) lentivectors were detected by qRT-PCR. b. Representative micrographs of wound healing assay in miR-205-5p-knockdown EN211 and EN307 compared with NC were shown. Images were acquired at 0 (white dotted line) and 48 h (black dotted line). Average migration rate per field was calculated. Scale bar, 20 μm. c. Representative micrographs of Transwell invasion assay in miR-205-5p- knockdown EN211 and EN307 compared with NC were shown. Average invasive cells per field were calculated. Scale bar, 50 μm. d. Representative micrographs of apoptosis assay in miR-205-5p-knockdown EN211 and EN307 compared with NC were shown. Average apoptosis rate per time was analysed. anti-205, anti-miR-205-5p. Error bars represent the mean ± SD of three independent experiments. **, P<0.01; ***, P<0.001. (TIF 4383 kb) [file 13287_2019_1388_MOESM5_ESM.tif]

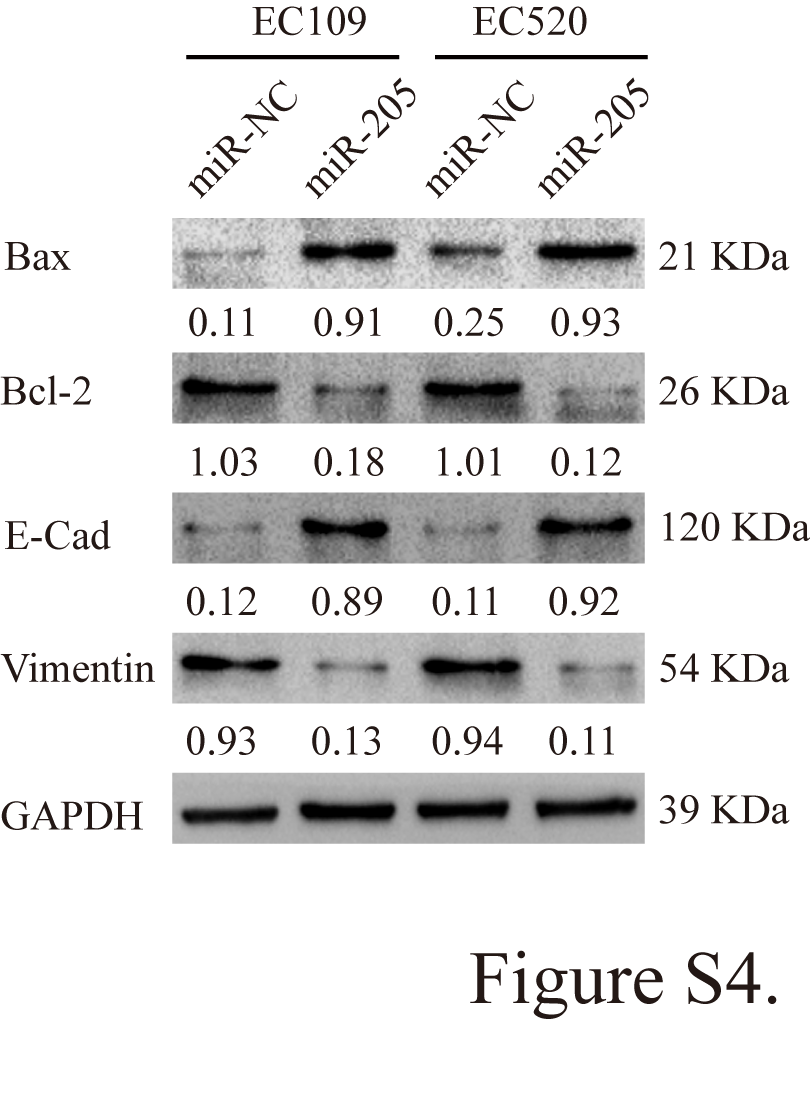

Supplement: Supplementary file 6 — Figure S4. The protein levels associated with migration, invasion and apoptosis in EC109 and EC520 cells transfected by lentivectors were detected by the western blot analysis, related to Fig. 2. miR-205, miR-205-5p. (TIF 937 kb) [file 13287_2019_1388_MOESM6_ESM.tif]

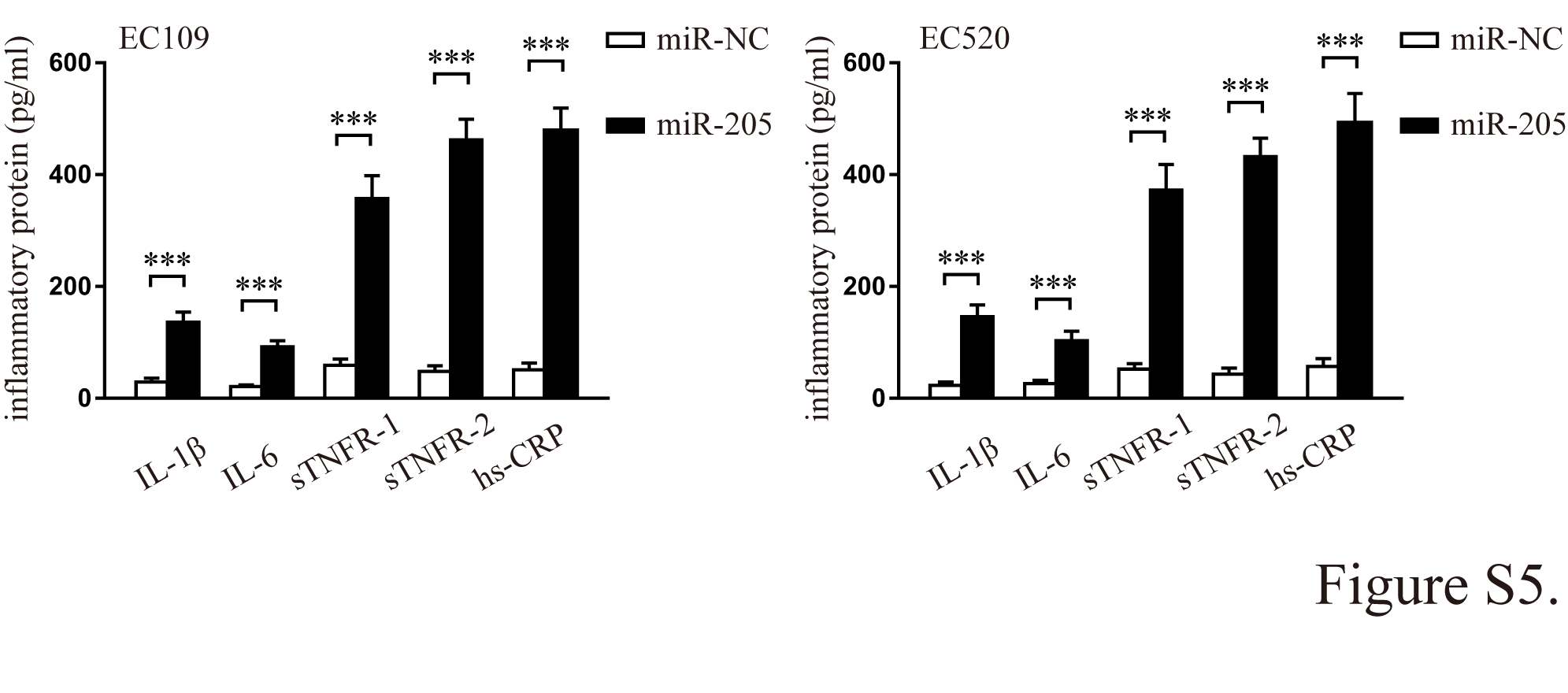

Supplement: Supplementary file 7 — Figure S5. The protein levels of interleukin-1 beta (IL-1 β), interleukin-6 (IL-6), soluble tumor necrosis factor α receptors 1 and 2 (sTNFR-1 and sTNFR-2), and high-sensitivity C-reactive protein (hs-CRP) in peripheral blood from animal model of endometriosis were detected by ELISA, related to Fig. 3. miR-205, miR-205-5p. Error bars represent the mean ± SD of three independent experiments. ***, P<0.001. (TIF 509 kb) [file 13287_2019_1388_MOESM7_ESM.tif]

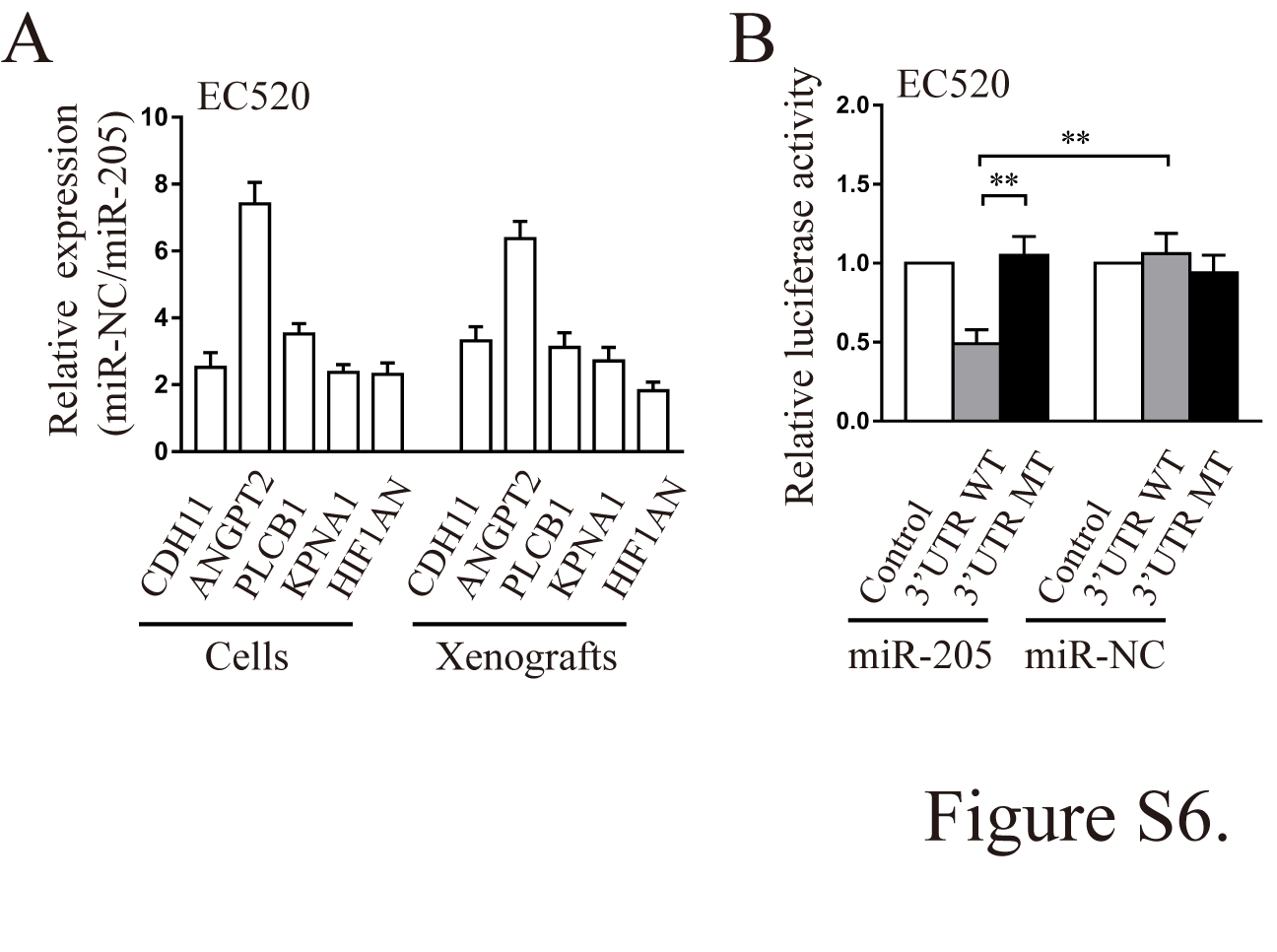

Supplement: Supplementary file 8 — Figure S6. miR-205-5p directly inhibited ANGPT2 expression via its 3’-UTR, related to Fig. 4. a. The RNA levels of CDH11, ANGPT2, PLCB1, KPNA1 and HIF1AN in cells and xenografts associated with miR-205-5p-overexpressed EC520 cells were analysed by qRT-PCR. b. The effect of miR-NC and miR-205-5p on the activity of the luciferase reporter containing either wild type (WT) or mutant type (MT) in EC520 were tested by dual-luciferase reporter assay. miR-205, miR-205-5p. Error bars represent the mean ± SD of three independent experiments. **, P<0.01. (TIF 483 kb) [file 13287_2019_1388_MOESM8_ESM.tif]

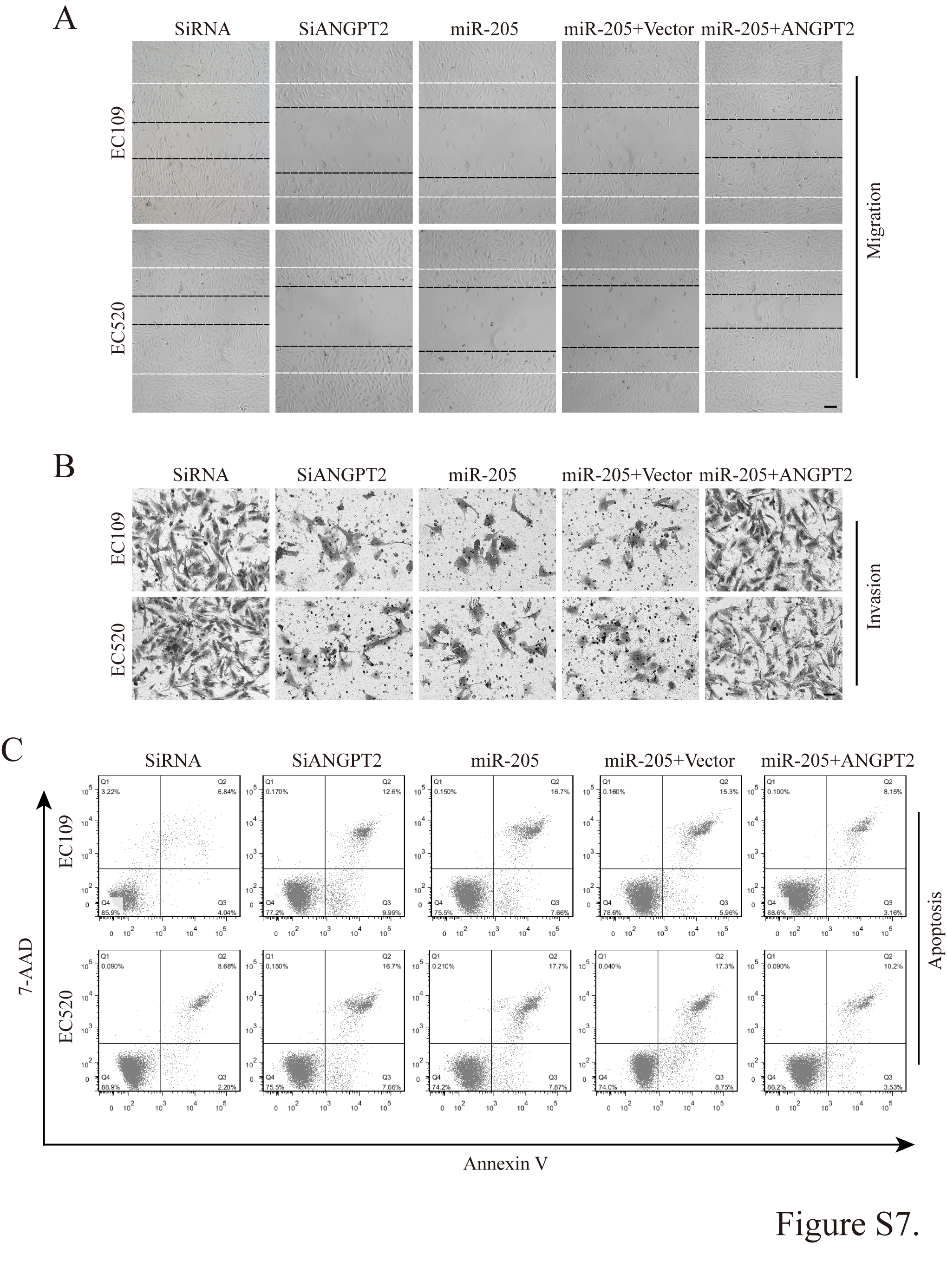

Supplement: Supplementary file 9 — Figure S7. The re-expression of ANGPT2 could rescue the suppressive effects of miR-205-5p in endometriosis, related to Fig. 5. a. Representative images of wound healing assay in EC109 and EC520 treated as indicated were shown. Images were acquired at 0 (white dotted line) and 48 h (black dotted line). Scale bar, 20 μm. b. Representative images of Transwell invasion assay in EC109 and EC520 treated as indicated were shown. Scale bar, 50 μm. c. Representative images of apoptosis assay in EC109 and EC520 treated as indicated were shown. (TIF 9701 kb) [file 13287_2019_1388_MOESM9_ESM.tif]
